# Editorial: Emerging technologies in imaging for pediatric heart conditions

**DOI:** 10.3389/fcvm.2026.1906983

**Published:** 2026-07-10

**Authors:** Harvey Ho, Liqun Sun

**Affiliations:** 1 Auckland Bioengineering Institute, Faculty of Engineering, University of Auckland, Auckland, New Zealand; 2 Department of Ultrasound, Women’s Hospital Zhejiang University School of Medicine, Hangzhou, China

**Keywords:** cardiac magnetic resonance, cardiovascular imaging, computed tomography angiography, echocardiography, electroanatomical mapping, fetal imaging, pediatric cardiology, speckle tracking

## 1 Introduction

Pediatric cardiovascular imaging is moving rapidly from anatomic depiction toward quantitative, multimodal, and decision-oriented care. This transition is especially important in children, where congenital and acquired heart conditions must be assessed across growth, where radiation and sedation should be minimized, and where small differences in anatomy or function may influence timing of intervention.

The 12 articles in this Research Topic, *Emerging Technologies in Imaging for Pediatric Heart Conditions*, illustrate this shift across fetal echocardiography, magnetocardiography, computed tomography angiography (CTA), cardiovascular magnetic resonance (CMR) 4D flow, speckle tracking echocardiography, multimodality imaging, Doppler-based diagnostic adjuncts, electroanatomical mapping, and imaging-linked clinical prediction. Collectively, they show that emerging imaging technologies are most valuable when they improve diagnostic confidence, refine risk assessment, or guide safer and more individualized treatment.

## 2 Ultrasound-based diagnosis and quantitative echocardiography

Several contributions demonstrate the continuing importance of ultrasound while expanding its diagnostic reach. Xie et al. showed that prenatal ultrasound diagnosis of fetal right aortic arch requires not only detection of the arch anomaly but also accurate subtype classification and assessment of associated cardiac, extracardiac, and chromosomal abnormalities, which are central to counseling and prognosis (Xie et al.). Liu et al. reported that abdominal aortic spectral Doppler combined with echocardiography can improve diagnostic sensitivity for pediatric aortic coarctation, particularly when standard echocardiographic views are limited (Liu et al.).

Other studies highlight the growing role of deformation and multi-parameter echocardiography. Lesmanawati et al. evaluated global longitudinal strain for early detection of doxorubicin-induced cardiotoxicity in pediatric cancer patients (Lesmanawati et al.). Goyal et al. applied two-dimensional speckle tracking to the ascending aorta, showing its potential for assessing arterial stiffness in repaired conotruncal anomalies (Goyal et al.). Yuan et al. used echocardiographic assessment to characterize preexcitation syndrome and left ventricular wall dyskinesia in children (Yuan et al.). Together, these studies reflect a broader transition from qualitative interpretation toward reproducible physiologic markers.

## 3 Cross-sectional, computational, and flow imaging

Advanced cross-sectional imaging is increasingly important when complex anatomy or physiology requires patient-specific quantification. Shah et al. developed a semi-automatic CTA-based method to estimate intramural length in anomalous aortic origin of a coronary artery, a high-risk feature relevant to surgical planning and sudden cardiac death risk stratification (Shah et al.). Nystrom et al. used CMR 4D flow to assess aortic and pulmonary wall shear stress in children with repaired tetralogy of Fallot, illustrating how flow-derived biomarkers may improve understanding of vascular remodeling and follow-up needs (Nystrom et al.).

These studies show how imaging datasets can be transformed into quantitative metrics that extend beyond visual interpretation. Such approaches may help reduce observer variability, support longitudinal assessment, and connect imaging more directly with clinical decisions.

## 4 Electrophysiologic imaging, mapping, and multimodality care

This Research Topic also broadens the meaning of cardiovascular imaging. Li et al. reviewed magnetocardiography, including fetal magnetocardiography, as a non-invasive and non-contact method for assessing cardiac electrical activity when conventional tools may be limited (Li et al.). Chan et al. examined radiofrequency ablation vs. cryoablation for pediatric atrioventricular nodal reentrant tachycardia in the era of three-dimensional electroanatomical mapping, emphasizing the value of map-guided, low- or no-fluoroscopy electrophysiology in children (Chan et al.).

Other papers emphasize that imaging rarely stands alone. Gao et al. identified risk factors and biomarkers associated with hemodynamically significant patent ductus arteriosus and ibuprofen treatment failure in premature twins (Gao et al.). Yuan et al. reported early predictors of fulminant myocarditis, including NT-proBNP, cardiac troponin I, alanine aminotransferase, and lactate (Yuan et al.). These studies reinforce the need to integrate imaging with biomarkers and clinical trajectories. Finally, Zhou and Chen reported multimodality imaging of congenital ventricular septal aneurysm with biventricular myocardial non-compaction, demonstrating the value of combining modalities in rare and complex pediatric disease (Zhou and Chen).

## 5 Future directions

Across the collection, three themes emerge. First, pediatric cardiovascular imaging is becoming more quantitative, with strain, Doppler indices, wall shear stress, CTA-derived morphology, and electroanatomical mapping serving as measurable biomarkers. Second, it is becoming more personalized, because many pediatric heart conditions require patient-specific assessment rather than generic anatomic labels. Third, it is becoming more integrated, combining ultrasound, CT, CMR, electrophysiology, computational tools, and biomarkers.

Future work should prioritize reproducibility, age-specific reference values, standardized protocols, multicenter validation, and outcome-linked imaging markers. Artificial intelligence and automation may improve workflow and measurement consistency, but pediatric datasets are often small and heterogeneous, making transparent validation essential. Equally important, emerging technologies should be implemented in ways that are accessible, cost-conscious, and attentive to radiation and sedation burden.

## 6 Conclusion

The studies in this Research Topic capture a field moving from imaging as depiction toward imaging as decision support. By aligning technical innovation with the needs of children, families, and multidisciplinary care teams, emerging pediatric cardiovascular imaging technologies can support earlier diagnosis, more refined risk assessment, safer interventions, and better lifelong outcomes.

